# Restricted by Measures Against the Coronavirus? Difficulties at the Transition from School to Work in Times of a Pandemic

**DOI:** 10.1007/s11217-022-09866-0

**Published:** 2023-01-24

**Authors:** Julian Valentin Möhring, Dennis Schäfer, Burkhard Brosig, Martin Huth

**Affiliations:** 1 Youth workshop Gießen gGmbH, Department of Sociology at the Distance University Hagen, Hagen, Germany; 2 Sigmund Freud Institute Frankfurt – Research institute for psychoanalysis and its applications, Frankfurt, Germany; 3 Family Psychosomatics, Dept. of General Paediatrics and Neonatology, Center for Pediatrics and Youth Medicine, Justus-Liebig-University of Giessen, Giessen, Germany; 4 Messerli Research Institute Vienna, Department of Philosophy, University of Vienna, Vienna, Austria

**Keywords:** Social deprivation, Vulnerability, Recognition, Emerging adulthood, Covid-19 restrictions, Sequence analysis

## Abstract

The paper begins with the prerequisite assumption that social deprivation is a fragile and porous category. Thus, our hypothesis is, that how people are affected by the restrictions against the spreading of the coronavirus is often discussed in far too general and simplistic terms. It is often taken as a given, that the virus and the restriction measures not only have caused severe difficulties for us all (due to social distancing, fear, affected health, etc.), but that the measures have exacerbated already previously existing forms of social deprivation. Therefore, it is assumed that marginalized groups are particularly affected by these effects of the pandemic. Two major German studies show the alleged effects of the relevant measures on adolescents and emerging adults (COPSY and FIBS) against the backdrop of social and economic deprivation; their results confirmed that social deprivation entails a higher degree of being affected by the pandemic.

However, this picture becomes thwarted by considering the results of 10 narrative interviews, that were conducted by us with emerging adults in a German vocational training institute between April and August 2021 and showed variegated forms of experiencing issues related to the pandemic. In our analysis, we used the method of objective hermeneutics. In this paper, we present our considerations by outlining two contrastive experiences with the Covid-19 pandemic, in order to highlight the divergent ways in which the pandemic has been experienced even among individuals who previously had been affected by social deprivation – at least at first glance – in similar ways. The conceptual lens through which we interpret these two examples is, firstly, the one of precariousness and precarity (Butler 2009). And secondly, the concept of recognition derived from Honneth’s pertinent theory and Butler’s account of recognizability will play a pivotal role. While Honneth’s approach allows us to emphasize the normative and existential role of the fulfilment of our basic desire for recognition and the detrimental implications of disregard, Butler provides us with the means to thoroughly analyse the socio-historical conditions of misrecognition and social invisibility. Moreover, Butler focuses specifically on the recognizability of precariousness (Honneth’s triad of love, being respected as a rights bearer and solidarity shows a different focus). Combining qualitative research on precarious live circumstances with a theoretical perspective on recognition can also be found in a stunning research on couples in atypical working relations (Wimbauer and Motakef 2019). Proceeding from these theories and ‘applying’ them to our empirical material, we seek to call into question a too generalizing and hegemonic picture of how people were affected by pertinent restrictions. Qualitative interviews are able to point us to significant differences in the experience of the restrictions.

In terms of an outlook, we suggest that a sensitization for previously excluded kinds of experiences forms a crucial basis for a less normalizing, that is, more inclusive account of interpreting the affectedness and needs of variegated social groups; the pandemic has influenced and altered structures of the recognition of vulnerability and, by the same token, made them more explicit. It thus is an occasion for a critical engagement with the recognizability of needs, deprivation, discrimination, and related phenomena.

## Introduction

The aim of this paper is to highlight some pitfalls that we detect in narratives regarding emerging vulnerabilities against the backdrop of the pandemic. Measures, such as social distancing, lockdowns, etc., have been described as the constitution of a shared “Covidworld” (Kidd and Ratcliffe 2020), in which we all experience a heightened vulnerability and diminished opportunities to pursue our projects. Yet, the determination of *particularly* vulnerable groups has been a crucial part of the measures (see below). Our hypothesis is, however, that the *recognition of vulnerability* within or through these narratives has developed in a biased and also too little differentiated way. What is more, the particular *experience* of one’s own vulnerability is often concealed either by generalizing narratives or by a focus on objective parameters of vulnerability, resp. risk. It is well known that, in Honneth’s account of recognition, being misrecognized is conceived of as specifically detrimental for the individual and its development (1996). Profound differences in education, social belonging, and career (and also connected economic situations) may well form an occasion for the emergence of misrecognition as basis for a corrupted relation-to-self. Honneth actually distinguishes between three basic versions of recognition: (a) (parental) love as a basis for self-confidence; (b) being respected as a rights bearer as a basis for self-respect; and (c) esteem/solidarity/the acknowledgment of valuable achievements as a basis for self-esteem (ibid., 107, 113, 118). All of them play a pivotal role for the individual’s integrity. Particularly – although not exclusively – the constitution of self-esteem is at stake when we consider difficulties in the transition from school to work. Being included in the labour market is a crucial prerequisite to be recognized as enacting one’s role as contributor to social cooperation; it forms a basis for respectability. Social deprivation can be conceived of as closely linked to a lack of opportunities to exert or participate in socially recognized activities. Particularly Young has pointed out that if the respectability of a person falls below a certain level, this forms a harm to the constitution of an appropriate relation-to-self (Young 1988, 284).

Honneth’s account shall be complemented with Butler’s reflection of recognizability. Basically, she proceeds from the distinction between *precariousness* and *precarity* (2009, 3 f.): While precariousness expresses the basic ontological vulnerability that all bodily beings share, precarity signifies the social constitution (and differential allocation) of vulnerability – often despite of, or even through, structures that are established in order to mitigate precariousness (see also Young 1988). Recognizability is the structural or societal sustenance of the visibility of vulnerability. Butler focuses mainly on the differential perceptibility treatment of different populations as being vulnerable and ailing in the aftermath of 9/11 (the Arab populations has been deprived of opportunities to grieve publicly; Butler 2004; 2009). Yet her conceptual lens can also be made fruitful for a closer analysis of variegated forms of structural deprivations and, more specifically, of the failure of generalizing conceptualizations of vulnerability to capture particular experiences of deprivation. Thus, our hypothesis is, that social deprivation is a fragile and porous category that cannot be determined once and for all.

Social deprivation became obvious in narrative interviews we conducted with emerging adults in 2017/2018, who were taking part in a vocational training measure in Germany. In our study, we explored the very experiences they made within this measure and found the concept of *emerging adulthood* (Arnett 2004) particularly useful to express their situation of being right in between in different aspects of identity: adolescence and adulthood, school and work (both can be brought in a relation to Honneth’s emphasis of the recognition of achievements), romantic partnership (as related to Honneth’s primal form of recognition rooted in a basic desire to be loved) and self-centrism (Möhring et al. 2021). We interviewed young people, whose educational record frequently contains school absenteeism and whose personal relationships especially to their families are strife-ridden (thus, a harm for these two forms of recognition became obvious). In comparison to the average population, the interviewees lacked significant opportunities for participation in social activities. This was due to a lack of economic and social capital. For instance, mobility and travelling were comparatively limited: None of them held a driving license or was travelling to other countries for at least several years.

Against the backdrop of the pandemic, we conducted another 10 interviews between April and August 2021 in the same setting as 2017/2018. While we all had to bear a deprivation regarding social events, travelling, social institutions that were shut down, etc. a widespread narrative has been – and still is – that the pandemic and the connected restrictions have affected people in precarious situations more intensely than the average population and that their social deprivation has been exacerbated. Opportunities to get included in social activities or in the labour market were further decreased while, what is more, their prevalence to get infected with the Covid-19 virus was significantly higher due to lack of information, confined living conditions, etc. (Bambra et al. 2021). However, as becomes apparent through the interviews, many people with lower socio-economic status regularly live with or in social deprivation, which resembles the situation we all experienced during the pandemic in several respects. What has been exceptional to an alleged average population is close to or equals the everyday experience of people who face social deprivation. Thus, they might even adjust more easily to experiences associated with the pertinent restrictions. Taking the subjective experience of people affected from socio-economic disadvantages into account, the standard narrative as outlined above can be called into question or at least has to be questioned regarding its scope and limits.

In addition, narratives that emerged throughout the pandemic tend to conceal deprivations that are not directly connected to the respective measures or implicitly justify the exacerbated divergencies in social capital, as if they were a matter of course during times like these. Therefore, it seems doubtable that it suffices to increase social cushioning, for instance, to the provision of adequate access to devices that enable remote learning. We suggest that this confronts us with a matter of recognition that cannot be tackled adequately with Honneth’s account. He highlights a specific dependency on recognition, which is significant for an analysis and critique of social exclusion, however, it is focused on love, respect for rights and the acknowledgment of contributions to social values. But what is at stake in our considerations is a misguided or biased recognition of vulnerabilities. We, therefore, suggest to enrich the understanding of recognition with the dimension of variegated forms of vulnerability. Butler’s account (2004, 2009) does not only provide us with the means to shift the focus from esteem to the recognition of (variegated kinds of) vulnerability, it also allows us to properly understand that recognition takes place within powerful structural conditions and limits. In order to understand the emerging adults’ situations, it seems indispensable to become sensitive and responsive to their specific vulnerabilities and how those vulnerabilities intersect with the way the pandemic has affected their recognizability.

Our aim is, first, to investigate how the pandemic and the restrictions affected their lives. Secondly, we seek to highlight the differences between our qualitative interviews and two broadly received quantitative studies about the effects of the Covid-19 pandemic on transitions from school to work. Notably, quantitative methods are rooted in a pregiven orientation of the questions, which are framed by structural conditions and limits that can be related to the notion of ‘recognizability’. The immanent limits of the scope and the socio-historical imbuement of the questionnaire are hardly perceptible if we just focus and rely on the data gathered by quantitative research. In contrast, using two particularly divergent qualitative interviews with young men, we seek to highlight differences in the very experience of the pandemic and the pertinent measures. These differences are sometimes not to be captured by dominant narratives. Subsequently, we are going to present a critical examination of the standard narrative of the Covid-19-restrictions as allegedly affecting especially people who are already confronted with social deprivation. Finally, it becomes apparent that social deprivation is a most important, yet fragile and porous category; its use must not go unreflected as this may result in inappropriate interpretations of particular vulnerabilities.

## Recognition, Normalization, and Particular Experiences

The reduction of difference through generalizing narratives builds an epistemic as well as an ethical issue. The relevant narratives that have emerged in the wake of our struggle with the virus, typical views on specific identities and connected vulnerabilities, serve as a matrix to understand particular cases and situations. Consequently, particular experiences are interpreted as cases of a general structure, and this very structure biased and biasing; that is, it allows to express experiences in a specific way and, by the same token, blocks other paths of understanding (Huth 2020). The recognizability bears a normalization that selects particular forms of vulnerabilities as socially relevant and/or visible. To be sure, the mentioned emergence of a ‘Covidworld’ (Kidd and Ratcliffe 2020) has increased the sensitivity for our shared (ontological) vulnerability. Yet, nevertheless, *specific* vulnerabilities in the face of the pandemic have been defined and made particularly visible – that is, the social recognizability of vulnerability has been altered and specified. Those who show recognizable vulnerabilities may well expect institutional and further support. The pandemic policies have proved that, as soon as someone has been recognized as belonging to a pertinent vulnerable group (e.g., due to the diagnosis of diabetes, age, Down’s Syndrome etc.), they could more easily benefit from the allocation of the first vaccines, medicines, and further protective measures. They are regarded as ‘typical atypical populations’ and, thus, as deserving special concerns. Those outside of these recognized minorities are often not only unrecognized, but even unrecognizable as having particular needs (Butler 2009, 9).^1^ Quite on the contrary, they may also have faced or face augmented stigmatization and exclusion. As will be illustrated with the interviews below, the socio-cultural particularity of a structure of recognizability turns recognition into a regulatory practice (Huth 2021), which sensitizes for *particular* sorts of vulnerability while concealing others. Butler emphasizes that the frames of recognizability are both a matter of perception and of policy (2009, 29); the hegemonic picture of those who deserve support is not only manifest in official institutions (including the medical system) but also in shared perspectives on members of a specific group. For example, while people with diabetes have been included into the category of vulnerable groups comparatively early, people with addiction hardly occurred as belonging to a risk group or risk patients.

As noted, large-scale quantitative studies on, for instance, the situation of families, children, adolescents, and emerging adults can be regarded as embedded in and to some extent as expression of these frames of recognition. The questions addressed are produced by socially approved views on what is significant during the transition from school to work, more specifically, how the transition may be affected by restrictions during the pandemic. At the same time, these studies achieve the highest visibility for effects of the Covid-19 pandemic and related restrictions of social life. But the question emerges how ‘extraordinary’ or ‘atypical atypical’ experiences can be addressed within such procedures. Forms of vulnerability that are not covered by powerful narratives (against the backdrop of the pandemic) might be misunderstood or bluntly invisible. The discursive and perceptual frames may lead us to miss the mark without being able to notice that. This is because they operate at the level of available concepts and unconscious dispositions to interpret, reflect and act (Rae 2022, 179 f.). Therefore, it seems that a normalization of vulnerabilities and connected social support is possibly reinforced precisely through empirical quantitative studies, which are understood as mere surveys producing descriptive insight to represent ‘reality’.

## Effects of the Covid-19 Pandemic on Adolescence and Emerging Adulthood

In what follows, we will describe the general orientation of two of these studies, emphasizing how they deal with socially deprived young people. Children and adolescents are frequently (albeit there is by far no unanimous consensus and albeit this emerged only after the first phases of the restrictions) seen as being among those groups who were affected the most by deprivations resulting from the Covid-19 pandemic. Ordinary life for pupils in regular as well as in vocational schools was interrupted due to measures of social distancing and school closures during lockdowns. The detrimental effects of social distancing for beings, who are conceivable as social beings in need for proximity and contact, have been analysed in detail (Carel et al. 2020); but this seems clearly exacerbated in emerging adulthood, as there is a particularly intense desire to interact and probe various sorts of staging oneself, but also to make experiences with love (as one basic form of recognition). A cross country study has shown (OECD 2021) that German schools were closed for a comparatively long time. Several scientific studies investigate into the effects the Covid-19 pandemic had on physical and psychological health in Germany; we will exemplify this with two major German studies on effects of the Covid-19 pandemic. While the COPSY study focuses on health-related problems, the FIBS study concentrates particularly on the measures’ effects on transitions from school to work.

### Example 1: Corona and Psyche (COPSY)

The COPSY-study (Ravens-Sieberer et al. 2021) investigates in the effects of the Covid-19 pandemic on the psychical and physiological health of children and adolescents in Germany. It was, moreover, directed at the identification of resources that supported children and adolescents to stay healthy (in an overarching sense) during the pandemic. Therefore, an online survey on psychological wellbeing was carried out in the time between May and June 2020 with children, adolescents and their families. All in all, *n* = 1586 parents of children aged 7 to 17 took part in the survey and *n* = 1040 children aged 11 to 17 years. State-of-the-art scientific instruments were used to understand health related quality of life, psychological oddities, anxiety, and depressive symptoms. The average age of the children who filled in the COPSY-survey is 14.3 years (51% female and 15.5% with a background of migration).

#### The Impact of Restriction by Measures Against the Coronavirus

71% of the interviewed children and adolescents felt burdened by the limitation of contacts during the pandemic. 65% experienced education as more exhaustive than before the pandemic. 27% reported an increase of conflicts at home and 37% of the parents reported that these conflicts with their children tended to escalate more often than before the pandemic. For 39% of the interviewed children and adolescents, contact restrictions were the reason for dissatisfaction with friendships.

40% of them reported a low-health related well-being measured with the KIDSCREEN-10-Index (Example item: “Have you felt sad?” or “Are you able to pay attention (concentrate well)?”). Before the pandemic, only 15% indicated such issues. Several psychosomatic symptoms increased with the implementation of restrictions to stop the spread of the virus, for instance, edginess (54% vs. 40%), sleep problems (44% vs. 39%), headaches (40% vs. 28%), dejection (34% vs. 23%), or stomach-ache (31% vs. 21%). All of these statistical data provide evidence, that the pandemic, generally, has increased the vulnerability of children and adolescents and had a negative impact on health-related quality of life, defined as an individual’s or group’s perceived physical and mental health over time (CDC, 2000).

Building on the COPSY-data, it seems possible to identify factors that increase risks concerning low health-related quality of life due to psychosomatic symptoms and an increase of anxiousness and depression (*n* = 106 children and adolescents), rooted in or reinforced by the Covid-19 pandemic, more specifically, by tensions within the family, parents with low education, a background of migration, or confined housing conditions below 20 square meters per person. In contrast, children and adolescents, who did not face comparable difficulties and who spent much time with their parents, reported a comparatively good health related quality of life. The authors of the COPSY-Study conclude, that the Covid-19 pandemic affected socially deprived populations more than the average population. They emphasize a need for prophylactic programs addressing populations with easy accessibility to secure and to enhance the mental and physical health of socially deprived children and adolescents.

### Example 2: Transitions to Work Life in Times of a Pandemic (FIBS)

Another study by the *Forschungsinstitut für Bildungs- und Sozialökonomie* (FIBS) (Dohmen et al. 2021) tackles the question of who amongst young people have been affected most by the decline of available apprenticeships in Germany. Their database derives from official sources on the transitions into apprenticeships, differentiated by school qualifications. The authors of FIBS estimate, that the Covid-19 pandemic has reduced the number of apprenticeships offered every year to a similar extent as the financial and economic crisis of 2007/2008. From 2008 to 2019, about 100,000 available apprenticeships disappeared. In addition, more than 50,000 apprenticeships have been lost since the beginning of the Covid-19 pandemic in Germany only in the time between March 2020 until February 2021. Getting access to the labour market obviously has become more difficult.

What is more, during the Covid-19 pandemic, high pressure emerged in the psychosocial environment of adolescents and emerging adults, caused by the insecurity of their parents’ working situation (possibly in-between job-loss and remote-desk) and by difficulties (e.g., home-schooling) of education for pupils in schools and participants in vocational schools. Diminished support and differences in the ability to work with modern educational tools further decreased the chances of obtaining a good school qualification or a college degree for those who belong to a lower social class (cf. Dohmen et al. 2021, 30). The problem of having decreased chances to get included in the labour market becomes more crucial for socially deprived adolescents and emerging adults during the crisis (ibid., 57 f.).

The studies outlined in this section clearly sensitize for the effects of the Covid-19 pandemic on adolescents and emerging adults. Problems associated with the Covid-19 pandemic have been examined by both studies; more specifically, the correlation between health-related, educational issues, and social deprivation became explicit. Both studies agree in that the burden for young people with a low socioeconomic status has been exacerbated by the pandemic (Dohmen et al. 2021, 50; Dohmen and Hurrelmann 2021, 13).

However, at this point, one runs the risk of understanding the Covid-19 pandemic as a general catalyst for existential difficulties related to social deprivation. Even though there is some plausibility in this claim, it would lead to a neglect of the variety of subjective experiences as well as the reification of social deprivation as if it would not be a contingent category with quite heterogeneous manifestations. The distanced view – neglecting the specific experiences of the individuals involved – on the situation of emerging adults in vocational training measures has already been criticized extensively (Hirschfeld 2020; Förster-Chanda 2020, Sveinsdottir 2018). What is more, some experiences that were formerly related to poverty only became part of our everyday life: being extraordinarily (compared to the situation before spring 2020) exposed to serious health threats; the fear of losing social capital and economic security; a lack of social contact; a lack of opportunities to exert leisure activities. Yet, the question emerges, how these issues became re-framed through the pandemic and how they relate to the recognizability of particular (and heightened) vulnerabilities within the shared social context. As noted, we doubt that the impact of the pandemic is reducible to a blunt reinforcement of previously existing forms of deprivation.

## Biographies in Transition

10 theme-centered narrative interviews (Küsters 2009) were conducted between April and August 2021. The background of this research project is the work of a psycho-social counselling service for young people, which is supported by the European Social Fond within the framework of the program “Qualification and Employment of young people in Hessen”. Emerging adults were asked to describe, how they experienced the past year in order to get a firm grasp of what they actually went through during the emergence of the pandemic. The interviewees were between 17 and 25 years old and took part in a qualification measure for at least several months. The interviews were held face to face. As became usual in everyday life during the Covid-19 pandemic, the interviewer and interviewee held their distance to each other, wore face masks and have been sitting next to an open window, however, a private atmosphere was possible. Our aim was to find out about their experience of the qualification measure as a biographical station and how the Covid-19 pandemic shaped this situation.

Notably, we found quite often small or even almost no changes in their everyday life (while, in contrast, the quantitative studies mentioned above confirmed the general assumption that relevant existential issues have been developed or exacerbated through restrictions such as social distancing). About half of the interviewees did not actively address the Covid-19 pandemic and only reported pertinent experiences upon the interviewer’s request. They usually emphasize (chronic) personal problems and family issues, which frequently affect their opportunities to succeed in the transition from school to work. The professional context of our research-group on psychosocial counselling for young people with problems at the transition from school to work helped us to understand that there are specific issues that can be traced back to a specific milieu, e.g., a significant lack of economic and social capital. However, clearly, a great range of different circumstances of life situations move young people into vocational training measures. There are large discrepancies between the everyday lives of interviewer and interviewee, that may influence their conversation. Presuppositions implicitly taken for granted may remain invisible when interviews are analysed insensitively or unreflectively. Therefore, we use the hermeneutical method of sequence analysis to make explicit, what otherwise would remain concealed (Oevermann 2000). Social barriers and dissents presented in the material are easily overlooked, if the material is accessed from the perspective of average routines and prevalent narratives. With this method, a text is examined for potential connections at each unit of meaning, if possible, without already applying a specific context. Only when a variety of connection possibilities have been thoughtfully considered, does the interpretation move on to the next sequence, and so on. Following this text work, a decision pattern emerges piece by piece, which is derived from the individual actions and action considerations described in the interview. With this procedure, insights into rules, regularities and routines of a social interaction can be gained even from small excerpts of a life practice.

In the following section, we will present two cases with a contrastive structure. The very structure of each case can be detected in similar forms of getting over everyday crises, which can be frequently found in their self-narrations. However, the contrasts manifest in the *modus operandi* towards the circumstances we all had and have to face during the Covid-19 pandemic, derived from objective hermeneutically analysed case structures (Kramer 2020). The personal data has been changed for all interviewees.

### Two Case Vignettes

In what follows, we will present two case vignettes in order to show quite contrastive ways of dealing with the pandemic against the backdrop of a comparatively precarious life situation. These two persons differ in activity, age, social background, migration, and occupation, but not in gender. We did not intend to focus on male interviewees only, we conducted 5 interviews with male and 5 with female participants. But the divergencies become most obvious as we compare these two cases.

#### But…yes! Diego between Fatalism and Orientation by Achievement

Diego (24) moved to relatives in Germany four years ago, together with his four siblings and his parents. The family came from Haiti, because his father could not find a job there and hoped for better opportunities in Germany to work as an architect. Diego holds a high school diploma from his home country, which is accepted as equivalent to a German secondary school certificate. Diego is fascinated by working with metal and tried to start a professional life with an apprenticeship in order to become an automotive mechatronic engineer. But he had to quit due to serious mental health issues. He nevertheless manages, with support from his parents, to undergo a therapy and again takes up a social-pedagogically supported apprenticeship at the vocational training institute.

But yes, after that I have had treatments with psychologist, psychiatrist, and pills (…) But now I’m always well and – yes. Do I have more stamina than before at work, for example, more concentration. Then – I can work more under stress than before, for example, where I didn’t know anything about what I had.

-Diego, Transcript 1

In this passage, we find his commitment to achieving goals. He seeks to succeed in coping with his social environment in a constructive way and, basically, for recognition and self-esteem in the sense that has been highlighted by Honneth (1996, 118). However, his psychic condition as well as a deep-seated insecurity (which will become more apparent in our considerations below) seem to thwart his attempts to satisfy his need for recognition.

In February 2020, his family moved from a rural area to a middle-sized town, and in May, he began to take part in a vocational training measure. He mixes up this story of moving to another town with the impact of the measures taken against the coronavirus.

Then people go out at night and scream and are drunk or something because of party or something. Suddenly everywhere is silence, you feel like, this would (laughing) be the end of the world, I don’t know.

But...yes. Mhm. Yes, it was very hard for me in the beginning, because I was used to living in a village (…) and everything was calm. And no, no bells or ambulance or drunken people.

-Diego, Transcript 1

In a later sequence, he reports how he deals with these new situations: living in a city and with the coronavirus:

“(…) there’s always something going on here, there’s always here and that and people gather, do sports or go partying. (…) But for me before, it was, oh, too many people or too much stress and I can’t do it, or I can’t manage it. But now I live with those through stress, somehow.”

“(…) Even though I was outside a lot in the summer last year, even though Corona and all that. And for me it was not so quite bad”.

-Diego, Transcript 1

In the interview, Diego’s performance is somewhat stiff; when reading somewhat between the lines, it becomes obvious, that he has a quite peculiar way to get along with the challenges of his life. If something goes wrong, he usually never blames the circumstances or the context for failures. It seems, as if his habituated style of attribution is directed at a high personal responsibility for his development: He takes his failure at the labour market for a personal mistake and for his (in his own view) bad performance.

Many of his sentences begin with “but…yes.” The insecure “but” is answered by himself with the focus on a “yes”, to deal with his insecurity, which is anchored in his personal experiences: moving from a village to the city and taking up an apprenticeship during the start of a global pandemic. His insecurity is probably rooted in his ongoing experience of being unsuccessful. For instance, his German mother spoke to him in her mother’s tongue from the very start, yet he answered in Spanish. Nevertheless, he started his German language course at the lowest level (A1). But also the experienced danger of becoming the victim of a street crime, which has been a constant threat to his everyday life in Haiti, may be a reason for his uncertain behaviour. As Young notes in her famous consideration of violence as one form of oppression – which seems particularly telling for Diego’s case –, the enduring normality of violence becomes particularly oppressive, as it imbues everyday life and forms an atmosphere of ongoing insecurity, which is incorporated (1988, 287). Diego shows such an imbuement in his uncertain appearance.

Insecurity was also at stake in his family when dealing with the corona virus; but here, Diego took the role of the one who can leave behind this insecurity (or suppress it). Diego’s parents didn’t want him to spend his time outside during the pandemic. They were afraid he could bring the virus to their house. But Diego spent his days outside, even during the lockdown, and met with his friends outdoors. This led to arguments with his parents and their fear of becoming infected with Covid-19. But after two of his brothers visited an event for kids, the whole family, except Diego, was infected. They all recovered in several weeks. Now the situation has changed, and the parents are afraid that he himself could become infected.

#### Tim: No Destination Without Attachment

Tim (18) spends his time playing video games all day. Only care for his most basic needs and the time he spends in the qualification measure interrupt this occupation. He lives in a flat included in a sheltered housing facility; he has broken up with his parents already years ago. His parents got divorced when he was a little boy. Living with his mother, he experienced domestic violence, and, as a consequence, he was raised within the children and youth welfare system.

My mother separated from my father. At that time in this women’s shelter thingy, I don’t know, she beat me there. Then the director said: I’ll call in the youth welfare office.

-Tim, Transcript 2

He went to his father five years ago, however, his residence did not last, because his welfare had been endangered due to neglect.

“Because I saw that my father is not exactly the best person. I’ve also tried to change him somehow, to have him look out for me and stuff like that. Eheh, brings nothing. With the man brings nothing. I said, yes, then I’ll go back to the home. It then took another two years until the youth welfare office then said, yes okay, we send the boy back to the home.“

“I don’t want to visit him either, I don’t want to see him either. I don’t want to have any contact with my family.“

-Tim, Transcript2

The other adolescents living with him are younger than him, which bothers him a lot. But he is going to leave town for a sheltered housing with other inhabitants more of his age. He is insecure, which profession he would be interested in. He changed from the vocational qualification measure into a program with lower thresholds to keep up with. He didn’t meet the requirements of the former measure, because he was absent too many times. He also has spent too much money for videogames and, therefore, even did not have enough to eat. The sheltered housing would put sanctions on him, if he doesn’t visit the vocational training measure. His language is rough, aggressive, and simple. He answers in short sentences and isn’t really willing to explain his situation himself in the form of a narration. It seems as if he does not really care about his future possibilities at the labour market or about closer social relationships: he seems quite disoriented. This holds also true for his perspective on the Covid-19 pandemic:

“Q: But here the pandemic, one could be infected.

A: Yes.

Q: Okay. It didn’t move you (laughing) or what?

A: Hm, I don’t really care. Pandemic isn’t interesting for me at all. If I have Corona, I have Corona. The worst that could happen is that I die.”

-Tim, Transcript 2

The fatalism in Tim’s answer is particularly impressive. Participants in qualification measures often tend to neglect themselves. Also, they frequently do not speak openly to workers in the field of social support. Nevertheless, the quote cited above reveals injuries Tim suffered in his childhood, which he reported earlier in the interview. He also states that his father did not care for him when he tried to live at his place; and that he also prefers not to have a relationship to his mother, either. His harsh words underline the disregard he has for himself as a result of the experience of a lack of primal recognition (being recognized in the sense of loved) within his family. It seems quite obvious that the development of his relation-to-self has been affected by growing up in these family conditions.

In spite of very different personalities, biographies, and ways of communicating, both interviews can be compared easily concerning the very experience of the restrictions against the Covid-19 pandemic. The divergencies shown in the interviews point at a wide range of possibilities of how the pandemic has been experienced by emerging adults.

**Table:** 

*Contrastive Cases*	
**Diego**	**Tim**
Background of migration	Autochthonous
In the midst of his 20s	At the end of his teenage years
Transition to apprenticeship	Transition to a measure to learn a daily structure
Lives with his parents and siblings	Lives alone
In a relationship	Single
Team sports	No sports
Outside / meeting people Professional psychological treatment for years	Inside / gaming Psychosocial consulting at the training measure
Restricted by measures in many ways	Nearly no difference due to the pandemic

In Tim’s case, experiences related to the pandemic hardly led to any behavioural changes. In contrast, for Diego the pandemic signified a profound privation. The contrastive opposition of the two cases shows clearly that isolation and minimal mobility can be a habituated mode of existence prior to the pandemic. Diego *suffered* directly from the restrictions. While it became actually easier for Tim to stay at home playing his videogames, Diego even started into a new chapter of his life (and highlights that he is glad that the effects on his parents’ and siblings’ health by Covid-19 were moderate). Due to his activity and to greater social capital, it is easier for Diego to cope with the challenges of life than for Tim, who faces many of them on his own and in a passive way. Yet in his very experience, Diego felt much more affected by the restrictions.

## Social Deprivation (en)during the Covid-19 Pandemic

The interviewees can be related to a particular, allegedly uniform milieu in terms of their educational situation, but they report quite different experiences; this also reveals that general narratives often tend to neglect the internal differences between individual experiences and biographies. The underlying recognizability of their particular vulnerability in the face of a “covidworld” shows its limits and a tendency to merge very divergent kinds of being-in-the-world. The contrast between these two cases precisely shows the heterogeneity of ways of being affected by the pandemic and pertinent measures also within a supposedly homogeneous social group (for the ethical and political implications of neglecting particularities of vulnerability through subsuming different individuals under one category see Huth 2020).

Notably, in both cases, the educational history is not significantly changed by the restrictions. Thus, the experiences of a problematic transition from school to work during the Covid-19 pandemic cannot be covered by the frame or narrative of increased burdens precisely due to the restrictions, which is, however, a conclusion of both COPSY and FIBS. The measured conditions of school to work transitions during the pandemic, such as emerging psychological problems or the decrease of apprenticeships are important. But if the analysis does not go any further, we get an incomplete, if not distorted, picture.

Also, in terms of practice, an adequate and responsive way of addressing affected individuals and their problems in the transition from school to work can hardly be rooted in such narratives. It can be seen as an insensitive attribution, if it is claimed that the consequences of the pandemic are the most intense in socially deprived populations (and for members of these populations in a similar or equal way). To be sure, the Covid-19 pandemic entailed a specific burden for all adolescents and emerging adults. For some time, almost everyone in Europe had to face social distancing, travel restrictions, shutdowns of cafés, theatres, restaurants, discotheques, etc.

This shows, that the assumption of an increased burden for socially deprived young people should be a starting point for the analysis, yet it cannot be conceived of as a result of sociological research, as it is over-simplifying and sometimes just inadequate when considering the very experience of these individuals. Moreover, crucially, it seems as if the major frame of ‘vulnerability due to/exacerbated by the pandemic’ tends to conceal forms of social deprivation, that are not as clearly interlinked with or affected by the restrictions. It becomes apparent that the structural recognizability of vulnerability (Butler 2009, 4) has been changed by the pandemic and relevant measures. New visibilities emerged (sometimes in fast succession), however, the narratives and frames that generate these visibilities are inevitably selective (ibid., 1, 51). Framing vulnerability means that some forms of deprivations or restrictions are particularly considerable while others are marginalized or even rendered invisible or insignificant. Moreover, these frames determine narratives that are generalizing, i.e., insensitive regarding divergencies. Yet, adolescents who did not regularly visit school before the pandemic are facing different consequences of the restriction in comparison to children who were well included in the school system. A reflexive recognition of the vulnerability constituted by the effects of the Covid-19 pandemic on emerging adolescents has to be sensitive for particular contexts and related experiences.

Considering deprivation and exclusion in society should further be aware of the limits on the part of the researcher in regards to comprehending actual experiences in a social milieu that is shaped by poverty. The narrative interviews we conducted make it possible to reconstruct some of these experiences, but some limits inevitably remain. For example, some interviewees called for protection against a possible misuse of the recordings; obviously, they did not fully trust the interviewers. They were hesitant to tell an unfamiliar interviewer about some of their experiences in detail (concerning problems with their family, sexuality, drugs and choosing a partner). This might be due to shame. The experience of shame may well emerge because of being marginalized and finding oneself outside of a dominant social normality. Shame can be conceived of as the downside of the struggle for recognition as analysed by Honneth (1996). In Young, this is related to the concept of respectability (1988, 284); the lack of this very respectability evokes shame on the part of the marginalized individual. Shame can be associated with a feeling of comparative inferiority (Vendrell Ferran 2022, 282). This may well be one reason for the fact that social boundaries are not easily crossed.

What is more, the self-narration may become a reproduction of or at least include the social gaze of allegedly normal ways to get along with this crisis, as well as how they refer to their family, relationship, work and education. Young highlights a ‘double consciousness’ emerging especially in oppressed or marginalized populations: “This sense of always looking at oneself through the eyes of others, of measuring one’s soul by the tape of a world that looks on in amused contempt and pity.“ (DuBois cited in Young 1988, 286). This might also entail a strangeness of the own experience and of own desires and preferences – and of their frustration. If the narratives include inadequate pictures of the relevant vulnerability, a distorted image of the self and their pandemic-related challenges may emerge in the affected individuals. In the vein of Butler (2009, 6), we can highlight that such a view is the outcome of a powerful historical *a priori* constituted through frames of recognition (recognizability) of vulnerability. The interrelation between personal narrative and social taboo could therefore result in future replications of the view, that this pandemic extremely lowered the chances to get an apprenticeship and was another burden to carry for those facing poverty and social tensions at home – even though we found almost no indication in our interviews, that they experienced a specifically increased burden due to the restrictions compared to the general population. Obviously, the narrative of an increased social deprivation due to restrictions against Covid-19 has only limited explanatory power to understand the situation of socially deprived people, including their own point of view (Ittner 2016, 24). Poverty induced corrosive disadvantage (Wolff and de-Shalit 2007, 10) may result in specific strategies of coping with the hardship of the pandemic (also revealing differences in conceiving illness in different social milieus).

Further, an official confirmation (e.g., a medical diagnosis) of a deficit is often mandatory to receive help from vocational training programs at the transition from school to work in Germany (Walther 2020). This leads to a further normalization of deviance or deficit; the confirmation makes a particular vulnerability recognizable and administrable. Yet, the scientific view tends to objectify subjective experiences and, again, constitutes an allegedly uniform social group. Further, concepts of maturity – as part of the recognizability of vulnerability – in relation to social deprivation might imply the danger to infantilize or paternalize affected individuals, even if the researcher or the social worker starts from the best intentions (see Huth 2020). This is confirmed by Graf and Schweiger, as they indicate: ”In fact, poor adults are often treated like children, in the sense that their choices and views are not seen as authoritative.” (2015, 106). It does not come as a surprise, that emerging adults – as but one example – try to protect themselves against such unintended forms of denigration by refusing to give (some) information about their experiences (as has already been pointed out). Research on the impact of the pandemic on various social groups should be aware of these obstacles and the biases on the part of those conducting this very research. To be sure, research on the pandemic is but one illustrative example here.

## Conclusion: On Patterns of Vulnerability Amid a Pandemic Situation

The Covid-19 pandemic gave rise to increased discussions on how to deal with social groups who are conceived of as vulnerable; basically, the determination of who is particularly vulnerable to the virus and/or to the restrictions has been a major subject of public and political debates. The course of the pandemic leads to worries about the chances of those who are already deprived of most important opportunities to self-realization and sources for recognition. In this paper, we have used the conceptual lens of recognition, in the vein of both Butler and Honneth, to emphasize not only difficulties at the transition from school to work, but also pitfalls in the research on populations that are conceived of as socially deprived. While it seems to be clear, that their vocational options are comparatively more decreased due to the consequences of the pandemic on the job market, it is far from clear that they experience more psychological distress and more restriction than the alleged average population. The recognizability of their vulnerability is ambivalent, as it implies, on the one hand, the visibility of the relevant vulnerability and the claim for social support, and on the other hand, the danger of paternalism, infantilization, generalization and stereotyping. As a consequence, there might be a lot of distrust in researchers, but also in those, who are part of the system of social support, in a way, because it is hard to change perspectives, if the other’s point of view seems exotic or unreliable. What is more, various kinds of vulnerability (and, thus, also vulnerable groups or individuals) may be excluded from the scope of recognizability; this may well lead to augmented forms of marginalization and social exclusion, which could even be beyond the scope of social sciences (as their conceptual lens might be too biased). As a consequence, we suggest to understand social deprivation (as well as vulnerability) as a fragile and porous category, which needs to be used with precaution.

Finally, in terms of an outlook, we would like to suggest that Butler’s concept of apprehension (2009, 4 f.) might be a helpful source in order to live up to the complexities of social life and of particular identities. Apprehension is a complementary concept to recognition/recognizability and emphasizes the possibility of going beyond the frames of recognition. Butler writes: “We can apprehend, for instance, that something is not recognized by recognition. Indeed, that apprehension can become the basis for a critique of norms of recognition. The fact is, we do not simply have recourse to single and discrete norms of recognition, but to more general conditions, historically articulated and enforced, of ‘recognizability’.“ (ibid., 5). Particularly when it comes to the issue of atypical vulnerabilities, the role of qualitative interviews might be the one of a sensitization (Liebsch 2018). What are the options of such a person, if he can’t stay a child, but has bad preconditions to become a part of the working force of his society? And if s/he is not “allowed” to work, does this mean that s/ he will not be recognized as an “adult” standing on her/his own feet by large parts of society in the future? What does this mean for intimate relationships and also possibly the offspring?

## Footnotes

1 Actually, Butler goes in these passages so far as to contend that some living beings are not even recognizable as subjects.
